# Impact of Fetal Exposure to Endocrine Disrupting Chemical Mixtures on FOXA3 Gene and Protein Expression in Adult Rat Testes

**DOI:** 10.3390/ijms24021211

**Published:** 2023-01-07

**Authors:** Casandra Walker, Annie Boisvert, Priyanka Malusare, Martine Culty

**Affiliations:** 1 Department of Pharmacology and Pharmaceutical Sciences, Alfred E. Mann School of Pharmacy and Pharmaceutical Sciences, University of Southern California, Los Angeles, CA 90089-9121, USA; 2 The Research Institute of the McGill University Health Centre, McGill University, Montreal, QC H4A 3J1, Canada; 3 Department of Medicine, McGill University, Montreal, QC H4A 3J1, Canada

**Keywords:** endocrine disruptors, EDC mixtures, in utero exposures, transcriptome analysis, testicular function, Leydig cells

## Abstract

Perinatal exposure to endocrine disrupting chemicals (EDCs) has been shown to affect male reproductive functions. However, the effects on male reproduction of exposure to EDC mixtures at doses relevant to humans have not been fully characterized. In previous studies, we found that in utero exposure to mixtures of the plasticizer di(2-ethylhexyl) phthalate (DEHP) and the soy-based phytoestrogen genistein (Gen) induced abnormal testis development in rats. In the present study, we investigated the molecular basis of these effects in adult testes from the offspring of pregnant SD rats gavaged with corn oil or Gen + DEHP mixtures at 0.1 or 10 mg/kg/day. Testicular transcriptomes were determined by microarray and RNA-seq analyses. A protein analysis was performed on paraffin and frozen testis sections, mainly by immunofluorescence. The transcription factor forkhead box protein 3 (FOXA3), a key regulator of Leydig cell function, was identified as the most significantly downregulated gene in testes from rats exposed in utero to Gen + DEHP mixtures. FOXA3 protein levels were decreased in testicular interstitium at a dose previously found to reduce testosterone levels, suggesting a primary effect of fetal exposure to Gen + DEHP on adult Leydig cells, rather than on spermatids and Sertoli cells, also expressing FOXA3. Thus, FOXA3 downregulation in adult testes following fetal exposure to Gen + DEHP may contribute to adverse male reproductive outcomes.

## 1. Introduction

Infertility is a global problem in which male factors have been found to account for nearly half of the cases [1,2]. The etiology of male infertility includes defects in sperm quality, low sperm count, ductal obstruction or dysfunction, or hypothalamic–pituitary axis disturbances [3,4]. Researchers have found lower sperm counts and decreased quality of semen in certain geographical regions, suggesting the influence of socioeconomic, nutritional, and/or environmental differences [1,5]. Decreased quality of semen has also been found to coincide with increasing incidence rates in male genital tract abnormalities such as cryptorchidism, a major risk factor for testicular cancer [6,7]. Additionally, infertility and male reproductive pathologies such as hypospadias and testicular cancer are on the rise in the Western world, and an estimated 10% of couples in the United States are classified as infertile [4].

The male reproductive system is one of the main targets of endocrine disrupting chemicals (EDCs), because of the requirement of sex hormones for its development and functioning, and the fact that many EDCs disrupt androgen and estrogen production and/or signaling [8,9,10]. While androgens produced by fetal testes drive the development of all male reproductive tissues, adult testes are dedicated to androgen and spermatozoa production, as well as regulating non-reproductive tissues. The testis is a complex and highly plastic tissue that comprises germ cells at different stages of development and several types of somatic cells. The main somatic cells are Leydig cells that produce androgens critical for the development and steady-state functions of the testis; Sertoli cells that regulate germ cell development and survival; peritubular myoid cells that contribute to interstitium components and germ cell regulation; and immune cells that maintain testis immune privilege and interact with other cell types.

EDCs are hypothesized to be causative agents of male reproductive disorders. Indeed, many studies, usually with individual EDCs given to pregnant dams at doses exceeding human exposure, have shown the disruptive effects of EDCs on male offspring reproductive functions, as summarized in the Endocrine Society’s statement review by Gore and colleagues [11]. EDCs can be natural compounds, such as genistein (Gen), a plant phytoestrogen found in soy products, baby soy formula, and vegetarian diets, or artificial compounds, such as 2-diethylhexyl-phthalate (DEHP), a plasticizer with anti-androgenic properties found in many consumer products. Gen acts as an estrogen-receptor agonist, and this mechanism of action forms the basis of its classification as a “phytoestrogen” [12]. Gen has been reported to be useful in the treatment of some cancers and chronic diseases by increasing apoptosis and differentiation. It has also been shown to alter early testicular germ cell development in rats [13,14,15] and to delay puberty in male primates [16]. Gen inhibits ATP-utilizing enzymes such as specific tyrosine kinases in vitro. Additionally, it has been found to have antioxidant effects and to inhibit angiogenesis. Of note is that some of these benefits only occur after consumption of a soy-rich diet. Moreover, genistein has been found to have low toxicity.

DEHP is a synthetic chemical used to increase the flexibility of plastics; it has been used for years in consumer goods such as household appliances, packaging, medical tubing, flooring, and other products [17,18,19]. Over 98% of the United States population has detectable levels of DEHP and its major metabolite MEHP in their urine. MEHP is also found in breastmilk. Studies have shown that there is a positive correlation between fast food consumption and DEHP levels in the body, and have stressed that people can easily get contaminated through common diets [20]. DEHP can reach the systemic circulation through ingestion and absorption by the skin. DEHP is an anti-androgenic compound that decreases Leydig cell production of testosterone in males [21]. It has also been shown to decrease Sertoli cell function and anogenital distance, an androgen-dependent process, in male rodents and humans [22].

Gen and DEHP have both been linked to male reproductive pathologies. However, the effects of EDC mixtures at environmentally relevant doses have not been well characterized. This identifies a need to evaluate the effects of perinatal exposure to Gen + DEHP mixtures at doses relevant to humans, to determine their impact on male reproduction. Our previous studies have found that in utero exposure to mixtures of Gen and DEHP (Gen + DEHP) at a dose mimicking the exposure level of the general population, and a higher dose mimicking that of more susceptible populations (such as hospitalized neonates), resulted in abnormal testicular development in adult (PND120) male rats [23]. Other investigators have reported similar responses to EDCs in germ cells from human and rat fetal/neonatal testes, validating the use of rat models to study the impact of EDCs on early germ cell development [7,24,25]. Since disrupting perinatal germ cells can hamper spermatogenesis and reproduction later in life, we hypothesize that fetal exposures to Gen + DEHP mixtures at doses relevant to humans impact the adult testes by disrupting the developmental program of key testicular cell types and altering their adult functions. Our goal is to identify the functional pathways altered by in utero exposure to Gen + DEHP mixtures, the testicular cell types in which these changes occur, and the mechanisms driving them that could explain the adverse reproductive effects observed [26].

## 2. Results

The present study used two transcriptomic approaches to establish DEG profiles and to perform a functional pathway analysis. First, we extended the analysis of microarray data collected during a previous study for a dose of 10 mg/kg/day [26]. Then, we performed a whole transcriptome RNA-seq analysis on additional samples to compare the effects of both 0.1 and 10 mg/kg/day treatments and to determine whether they disrupted the same molecular pathways and genes (See Flow-Chart in Materials and Methods).

### 2.1. Sertoli and Germ Cell Functional Pathways in Adult Offspring Are Altered by In Utero Exposure to Gen + DEHP Mixtures

We imported the list of 1184 differentially expressed genes affected by Gen + DEHP at a 10 mg/kg/day dose into the Database for Annotation, Visualization and Integrated Discovery (DAVID) linked with the Kyoto Encyclopedia of Genes and Genomes (KEGG). The KEGG pathway revealed that many DEGs were related to Sertoli and germ cell pathways (Table 1). Among those, the Hippo signaling pathway is a conserved growth control pathway that plays a role in regulating proliferation of various cell types and has been found to be important for Sertoli cell function [27]. The Wnt pathway has been found to promote spermatogonial stem cell maintenance by suppressing apoptosis via the beta-catenin pathway [28]. Beta-catenin mRNA and protein are predominant in the seminiferous tubules of fetal mice and beta-catenin has been found to be abundant in Sertoli cells. Retinoic acid is critical for spermatogenesis and male fertility [29]. Additionally, retinoids are important for proliferation and differentiation of type A spermatogonia and spermiogenesis. Adherens junctions are found between Sertoli cells and Sertoli and germ cells, ensuring nutrient transfer from Sertoli to germ cells, and proper movement of germ cells from the basement membrane to the lumen. The MAPK pathway has been found to regulate the dynamics of tight junctions and adherens junctions and is involved in the proliferation and meiosis of germ cells [30]. These data suggest that in utero exposure to Gen + DEHP affects cells within the seminiferous tubules of the testis, Sertoli and germ cells.

### 2.2. Forkhead Box A3 (Foxa3) Is the Most Downregulated Gene in Adult Testes from Rats Exposed in Utero to Gen + DEHP Mixtures

**Figure 1:**
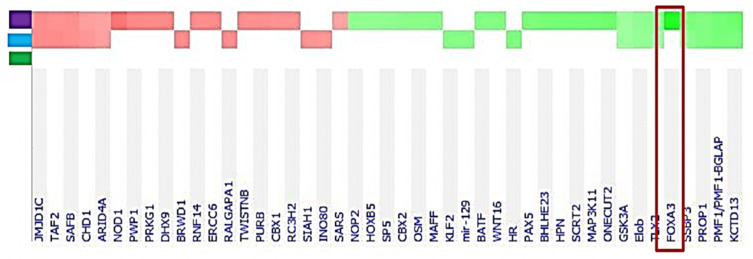
*Foxa3* is significantly decreased in rat testes exposed in utero to Gen + DEHP mixtures, but not by Gen or DEHP alone. Ingenuity pathway analysis (IPA) of the microarray data searching the term “transcription” identified *Foxa3* (red box) as downregulated in testes from adult rats exposed in utero to 10 mg/kg/day Gen + DEHP (*p*-value ≤ 0.05). Treatments: Gen, green; DEHP, blue; Gen-DEHP, purple. Expression changes: Red, upregulation and green, downregulation.

Next, we examined changes in the expression of genes identified as differentially expressed by conducting a microarray analysis in rats exposed in utero to a Gen + DEHP mixture at 10 mg/kg/day as compared with control rats. Among 1184 DEGs in the Gen + DEHP-exposed rats, the transcription factor forkhead box A3 (*Foxa3*) (also called hepatocyte nuclear factor 3γ) was the most downregulated gene, positioning *Foxa3* as a long-term testicular target gene of fetal exposure to EDC mixtures (Table 2). Additionally, it was the only transcription factor on this list. Interestingly, CYP11A1, the cytochrome P450 metabolizing cholesterol to pregnenolone, the first step in steroid formation, was significantly upregulated. Using ingenuity pathway analysis (IPA), next, we compared the effects of fetal exposure to Gen and DEHP alone to those of the mixture at 10 mg/kg/day, using the search term “transcription”. This analysis determined that *Foxa3* was highly differentially expressed only in in utero Gen + DEHP exposed rat testes (Figure 1). Whereas *Foxa3* was significantly downregulated by 62% of control values by Gen + DEHP fetal exposure (−2.60-fold change, *p* = 0.0014), it was only decreased by 30% of controls in rats exposed to DEHP (−1.42-fold change, *p* = 0.0271), and not significantly altered by fetal exposure to Gen (−1.12-fold change, *p* = 0.0816). Among these genes, only a few genes showed significant changes following fetal exposure to DEHP: Tmem249 (−1.31-fold change, *p* = 0.007), Tmem210 (−1.45-fold change, *p* = 0.007), Krt86 (−1.43-fold change, *p* = 0.033), and Pkmyt1 (−1.69-fold change, *p* = 0.005). No genes were altered by fetal exposure to Gen alone. Regarding the 10 most upregulated genes, Cyp2a1 was increased to a lesser extend by fetal exposure to Gen alone (2.99-fold change, *p* = 0.047) than by the mixture, and there was no significant increase in rats exposed in utero to DEHP alone. Cyp11a1 was slightly increased by GEN exposure (1.29-fold change, *p* = 0.001) but not significantly changed with DEHP alone. None of the other genes upregulated by the mixture were altered by Gen or DEHP alone. FOXA3 has previously been reported as the only FOX A family member identified in testes. More specifically, it has been found to be expressed in Leydig, Sertoli, and germ cells. A study by Behr and colleagues, in 2007, found that mice that were homozygous or heterozygous for the FOXA3 null allele exhibited reduced male fertility secondary to increased germ cell apoptosis [31]. These data provide further rationale to exploring the long-term effects of fetal EDC exposure on adult phenotypes, such as FOXA3 downregulation on testicular functions.

**Figure 2:**
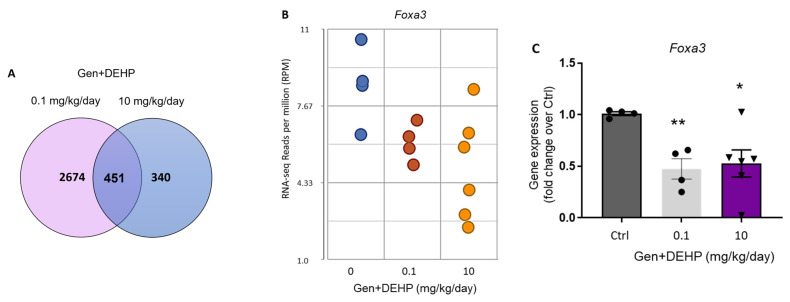
*Foxa3* expression is decreased in rat testes exposed in utero to Gen + DEHP mixtures: (**A**) Venn diagram of DEGs for both exposure doses, from Partek Flow analysis of RNA sequencing data; (**B**) RNA-seq analysis of *Foxa3* expression performed on rat testes exposed to 0.1 (Red dots) or 10 mg/kg/day (Orange dots) of Gen + DEHP mixture as compared with control rats (Blue dots). *N* = 4 rats from independent dams; (**C**) qPCR analysis assessing *Foxa3* mRNA expression in rat testes exposed to Gen + DEHP mixtures. *N* = 4 to 6 rats from different dams. Significance p values: * *p* ≤ 0.05; ** *p* ≤ 0.01.

The whole transcriptome RNA sequencing analysis (RNA-seq) conducted on additional samples revealed that there was a higher number of differentially expressed genes at the lower dose of 0.1 mg/kg/day as compared with the higher dose of 10 mg/kg/day for Gen + DEHP (Figure 2A). The RNA-seq showed that *Foxa3* was decreased, similarly to the microarray data, with overlapping values between GEN + DEHP doses of 0.1 and 10 mg/kg/day (Figure 2B). Similar data were obtained by qPCR analysis, showing significant decreases in *Foxa3* at both doses (Figure 2C). These results suggest non-monotonic effects of fetal exposure to EDC mixtures, and the further need to evaluate them at doses below currently documented NOAELs.

**Figure 3:**
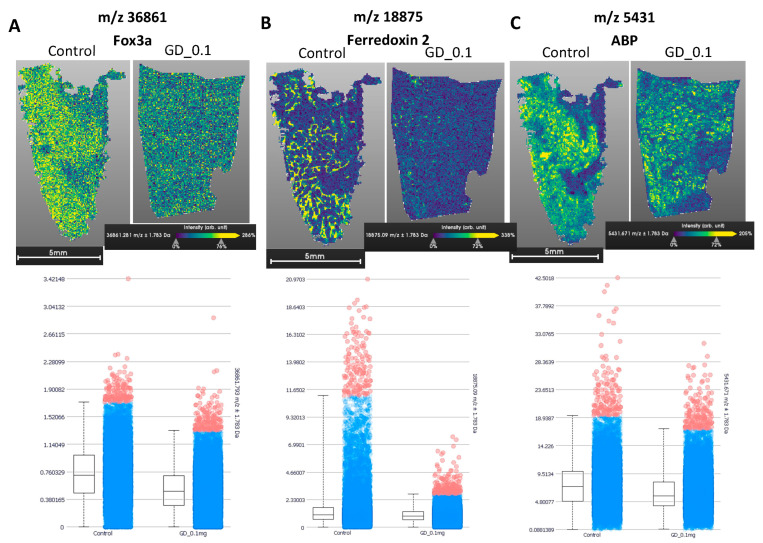
MALDI imaging mass spectrometry reveals that FOXA3 protein is decreased in adult testes after in utero exposure to Gen + DEHP mixture. Proteins were identified according to their mass in frozen sections of PND120 rat testes treated with vehicle (control) and Gen + DEHP mixture (GD) at 0.1 mg/kg/day, analyzed by MALDI IMS: (**A**) Foxa3; (**B**) Ferredoxin 2; (**C**) Androgen-binding protein (ABP). For each protein, the left panel shows the intensity of protein signal, with yellow representing the highest intensity; while the Box plots in the right panel show the quantification of relative signal intensity in testis sections, normalized by surface unit.

To assess global FOXA3 protein levels in testes, we used MALDI imaging mass spectrometry (MALDI IMS) to quantify the amount of FOXA3 protein in frozen adult testis sections from control rats and in utero Gen + DEHP exposed rats as compared with ferredoxin 2, another Leydig cell protein, and to the Sertoli cell marker, androgen-binding protein (ABP). The protein levels of Foxa3 and Ferredoxin 2 were both reduced in testes from rats exposed in utero to 0.1 mg/kg/day Gen + DEHP mixture, as shown by decreases in yellow signals on the sections and the histograms quantifying the normalized signal intensity by surface unit of sections as compared with the control rats (Figure 3A,B). In contrast, the intensity of the Sertoli cell protein Abp was strong in the control and EDC-exposed samples (Figure 3C). These results confirmed Foxa3 as a critical long-term testicular target of fetal exposure to Gen + DEHP, in agreement with the mRNA data.

### 2.3. FOXA3-Interacting Genes Are Differentially Expressed in Testes from in Utero Gen + DEHP-Exposed Rats

Our next goal was to identify genes regulated by FOXA3 within the testes. First, this was accomplished by running an in silico search of the IPA database for DEGs predicted to be targets of FOXA3 in the literature. Noticeably, the two doses of Gen + DEHP mixtures identified different putative targets of FOXA3, suggesting that doses with a 100-fold difference did not act on the same molecular mechanisms (Table 3A,B). Interestingly, the lower dose mainly downregulated genes, including the transcription factor Foxa1 and other DNA-interacting proteins (Table 3A), whereas the higher dose induced gene upregulation (Table 3B).

Since we used whole testis extracts, it was important to determine the specific cell type(s) where FOXA3 was decreased. We used TRANSFAC, a transcription factor database, to further explore FOXA3-interacting genes. This analysis showed that FOXA3 target genes were located in Leydig, Sertoli, and germ cells, with many of these genes found in Leydig cells (Table 3C). These data suggest that FOXA3 may be significantly decreased in Leydig cells of the testes and that this decrease could affect FOXA3 target genes and disrupt Leydig cell functions, including steroidogenesis. Among the genes listed in Table 3A,B, none of the genes were significantly altered by in utero treatments with 10 mg/kg/day of Gen or DEHP alone, although Cox4i2 and Khdrbs2 showed decreasing trends of −2.78-fold change (*p* = 0.083) and −2.07-fold change (*p* = 0.312), respectively, in rats exposed to DEHP alone. These data further highlighted the unique position of FOXA3 as a long-term target of in utero exposure to Gen + DEHP mixtures (Supplemental Table S1).

### 2.4. Fetal Exposure to Gen + DEHP Mixtures Decreases the Protein Expression of FOXA3 in Adult Testicular Interstitium

**Figure 4:**
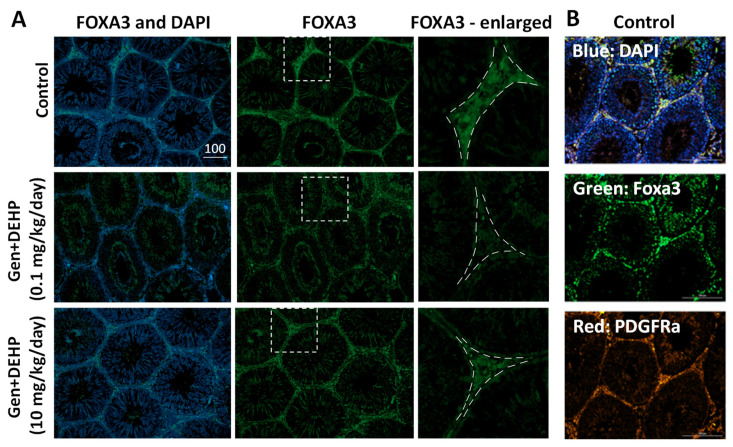
FOXA3 protein expression is decreased in testicular interstitial cells from adult rats exposed in utero to Gen + DEHP mixtures: (**A**) FOXA3 IF signal in testes from control rats and from rats exposed to Gen + DEHP mixture at 0.1 or 10 mg/kg/day. Left panel, merged IF images of FOXA3 (green) and DAPI nuclear staining (blue); middle column, FOXA3 IF alone; right column, magnification of white dotted inserts indicated in middle column; (**B**) IF colocalization of FOXA3 (green) and PDGFRa (red) in control rat testes. Representative photos are shown. Same scale in µm used for left and center columns in panel A, and in all pictures of panel B.

A study by Behr and colleagues reported that mice that were homozygous for the FOXA3 −/−null allele were infertile [31]. Another study found that FOXA3 bound to PDGFRa in Leydig cells, which was necessary for Leydig cell differentiation and embryonic development [32]. To determine in which cell types FOXA3 protein was downregulated, we examined the protein expression profiles of FOXA3 in testes of adult rats exposed in utero to vehicle, 0.1, or 10 mg/kg/day of Gen + DEHP mixtures by IF analysis. FOXA3 gave positive signals in the interstitium of control rat testes (Figure 4). Positive FOXA3 signal was also seen in germ cells within the seminiferous tubules in control samples, in agreement with published data. Although FOXA3-positive interstitial cells were visible in all rat testes, suggesting no change in cell numbers, the signal intensity of FOXA3 in these cells was reduced by fetal exposure to EDC mixtures, indicating that these treatments did not prevent the formation of adult Leydig cells, but rather altered their protein profiles (Figure 4A). FOXA3 levels were also decreased in seminiferous tubules of testes from rats in utero exposed to Gen + DEHP mixtures at both doses, while DAPI nuclear signal showed that germ cells were present in these tubules (Figure 4A). Quantification of FOXA3 relative signal intensity for several images per sample using ImageJ after grey scale conversion showed that the FOXA3 relative signal intensity in interstitial cells was reduced by 27% of control values for rats in utero exposed to the lower dose, and was only reduced by 12% for the higher dose (244.7 ± 9.9 in control rats, 179.0 ± 8.9 for Gen + DEHP 0.1, and 216 ± 5.9 for Gen + DEHP 10). The reduction at the low dose was similar to that measured by MALDI IMS. Inside the seminiferous tubules, FOXA3 relative signal intensity was reduced by 29% and 27% of control values for the lower and higher doses, respectively, (251.6 ± 14.6 for control rats, 178.5 ± 10.6 for Gen + DEHP 0.1, and 184.7 ± 10.8 for Gen + DEHP 10). IF analysis in control samples of FOXA3 and PDGFRa, both expressed in Leydig cells, showed that the two proteins colocalized in the interstitium (Figure 4B) [31]. The colocalization of FOXA3 and PDGFRa, and the appearance of FOXA3-positive cells in the interstitium, suggest that the adult interstitial cells in which FOXA3 expression was reduced by fetal exposure to the EDC mixtures were Leydig cells. Thus, FOXA3 expression in adult testes was altered by fetal exposure to the EDC mixtures both in interstital cells—most likely Leydig cells—and germ cells, with fetal exposure to the lower dose having stronger effect than the higher dose in interstitial cells, but similar effects in germ cells.

### 2.5. Gen + DEHP Mixtures Decrease the Expression of Genes/Protein Related to Steroidogenesis

We further used IPA to identify genes in our dataset that were related to the search term “steroidogenesis”, the main function of Leydig cells. All genes identified were significantly down- or upregulated, at least two-fold changes, in adult testes by fetal exposure to 0.1 mg/kg/day Gen + DEHP mixture as compared with the control rats (Table 4).

Growth hormone releasing hormone (GHRH) has been reported to be present in interstitial cells and germ cells in rat testes [33]. The same study also found that GHRH secreted by Leydig cells in adult rats could stimulate cAMP formation via induction of luteinizing hormone (LH), and that it could regulate Sertoli cell function. Cytochromes 7B1, 7A1, and 2R1 were also found to be decreased in the study. Cytochrome P450s in the testes are important for metabolizing cholesterol into testosterone [34]. Cytochrome P450 family 7 subfamily B1 has been found to regulate 11 beta-hydroxysteroid dehydrogenase 1 (11b-HSD1) in rat Leydig cells [34]. CYP7A1 and CYP2R1 are also part of the cholesterol synthesis network. Translocator protein TSPO binds cholesterol and transports it to the inner mitochondrial membrane for steroid biosynthesis to occur [35]. The family of apolipoproteins (i.e., APOA1) are also responsible for cholesterol transport [36]. Forkhead box A1 (FOXA1) is a member of the forkhead box A family that has been found to bind to the androgen receptor in the prostate [37]. From these genes, only *Apoa1* was significantly increased following fetal exposure to both doses of Gen + DEHP mixtures. The other genes were significantly altered only at the lower dose. None of these genes were significantly altered by fetal exposure to Gen or DEHP alone at the 10 mg/kg/day by RNA-seq analysis (Supplemental Table S2). These data suggest that steroid biosynthesis is affected in adult Leydig cells of the testes, while a reduction in GHRH could also suggest disturbances within the hypothalamic-pituitary axis, thus, affecting testosterone feedback. Since these are lists of FOXA3 target genes, these data suggest that a reduction in FOXA3 results in a reduction of GHRH, CYP7B1, CYP7A1, CYP2CR1, TSPO, FOXA1, and HSD17B, while the opposite may occur for APOA1.

**Figure 5:**
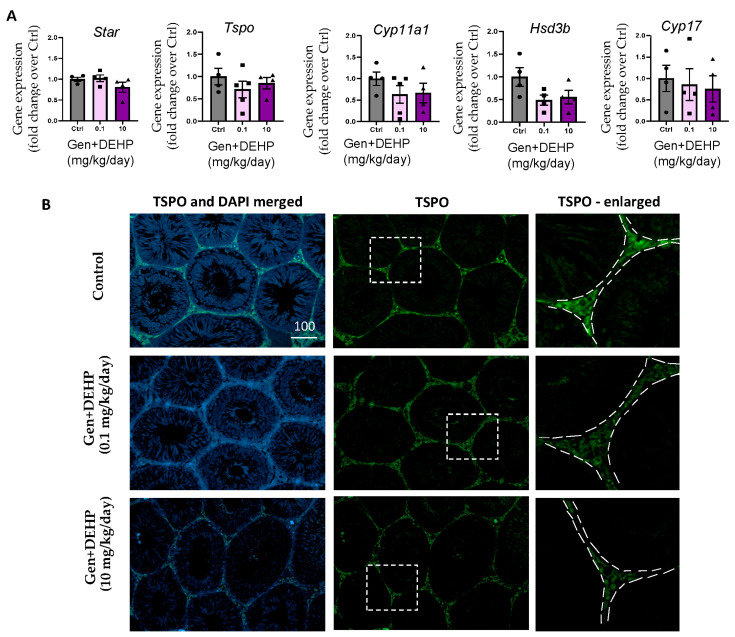
Fetal exposures to Gen + DEHP mixtures affect adult steroidogenesis-related genes: (**A**) qPCR analysis of steroidogenic genes in control (Crtl), Gen + DEHP mixtures at 0.1 and 10 mg/kg/day, excluding infertile rats. *N* = 3–5 per treatment; (**B**) protein expression of TSPO identified by immunofluorescence (green) showing reduced protein expression in testes from rats exposed in utero to 0.1 and 10 mg/kg/day of Gen + DEHP mixtures as compared with control rats. Representative photos are shown. Same scale in µm used for left and center columns in Figure 5B. Pictures in the right column are enlarged views of the white dotted area in pictures from center column.

We also examined the mRNA levels of several key genes of the steroidogenic cascade by qPCR in the testes of control rates and Gen + DEHP-treated rats after excluding infertile rats, to identify steroidogenic genes that might be related to less serious adverse phenotypes than infertility in in utero EDC exposed rats. We found that *Tspo*, *Hsd3b*, *Cyp11a1,* and *Cyp17* showed decreasing trends in rats exposed to 0.1 mg/kg/day, with *Hsd3b* decrease reaching close to significance with a *p*-value of 0.0504 at dose 0.1, and a *p*-value of 0.0713 at dose 10 (Figure 5A). Additional studies will be needed to determine whether these alterations are due to FOXA3 mediation or are a result of other disturbances. TSPO is a well characterized protein that is critical for steroid biosynthesis in testes [12]. While the qPCR analysis showed a decreasing trend in *Tspo* for fetal exposure to the dose of 0.1 mg/kg/day, TSPO protein levels showed some decreases in testes of adult rats exposed in utero to both doses of Gen + DEHP mixtures (Figure 5B). Quantification of the relative signal intensity of TSPO in several pictures/samples using ImageJ after grey scale conversion showed that TSPO protein levels were decreased by 14% in interstitial cells of rats exposed to the lower dose of mixture, and only 9% for the higher dose of EDC mixture (224.5 ± 1.5 for control rats, 193.7 ± 3.9 for Gen + DEHP 0.1, and 205.0 ± 4.9 for Gen + DEHP 10), while there was no noticeable change in the tubules, in which spermatogenesis was visible. The small changes in TSPO expression in the treated rats as compared with the significant changes in mRNA-seq analysis performed on different sets of rats likely reflect the variability in the adult reproductive phenotypes observed in in utero-treated rats, where 25% and 38% had abnormal testes, including Sertoli-only tubules, and/or small litters at both doses of Gen + DEHP mixtures, respectively, and 13% were infertile for both doses [26].

Taken together, these data suggest that FOXA3 may bind to and affect the transcription of several genes important for steroid biosynthesis, ultimately affecting steroidogenesis in testes. This is consistent with our previous study using the same treatments, in which we found that circulating testosterone levels were significantly reduced in adult rats exposed in utero to 0.1 mg/kg/day of Gen + DEHP mixture [26]. This further suggests that *Tspo* gene expression may be regulated by FOXA3, in a direct or indirect manner, but not in all in utero EDC-exposed rats.

### 2.6. Identification of FOXA3 Target Genes in Rat Testes by ChIP-Seq Analysis

We used ChIP-seq analysis to identify potential gene targets of FOXA3 in adult control rat testes (Table 5), and then examined the expression levels of these genes in the RNA-seq dataset. Only one gene, *Phlpp1*, showed a significant change, in that case a 20% increase, in the testes of rats exposed to 0.1 mg/kg/day of Gen + DEHP mixture, while *Phlpp1* showed a 68% increasing trend for the 10 mg/kg/day dose (Table 6). *Tmeff2* was another gene presenting increasing trends of 50% and 55% above control levels for 0.1 and 10 mg/kg/day of Gen + DEHP mixtures, respectively. In contrast, *Cxcl13* expression showed a decreasing trend in the testes of rats exposed to 0.1 mg/kg/day of Gen + DEHP mixture. Since a RNA-seq transcriptome analysis is performed on total testis RNA, and FOXA3 is expressed not only in Leydig cells, but also in germ and Sertoli cells, it is possible that adult FOXA3 expression might be differentially affected by fetal exposure to Gen + DEHP mixtures and might have different target genes in these cell types, potentially masking its effect in Leydig cells. Further studies are needed to examine this possibility. None of these genes were significantly altered in adult testes by fetal exposures to Gen or DEHP alone (Supplemental Table S3).

## 3. Discussion

DEHP, a phthalate plasticizer used in many commercial products and medical devices, and Gen, a phytoestrogen abundant in baby soy formula and vegetarian diets, are among the hundreds of chemicals with potential EDC activity to which we are exposed in our daily life. Our previous studies have shown that in utero exposure to Gen + DEHP mixtures increased the rates of infertility and abnormal testis development, altered gene expression, and induced inflammatory processes in adult (postnatal day (PND) 120) rats, differently from exposure to Gen or DEHP alone [26]. In the present study, we focused on the effects of fetal exposure to Gen + DEHP mixtures, with the goal of identifying long-term alterations of functional pathways and genes in adult rat testes, to gain insight into the etiology of the observed reproductive phenotypes in adult testes. In this study, we used mixtures of Gen and DEHP given at doses equivalent to levels measured in humans, to increase the chance of identifying long-term target pathways that could be meaningful for humans.

We found that in utero exposure to Gen and DEHP mixtures altered functional pathways related to Sertoli, germ, and Leydig cell development and function, in adult rat testes. More importantly, the transcription factor FOXA3 was downregulated in adult testes exposed to the mixtures as fetuses, but not to the same extent by fetal exposure to Gen or DEHP alone. FOXA3 is a transcription factor that has previously been identified in Leydig, Sertoli, and germ cells and is critical for testicular function [31,32]. In the present study, we showed that *FOXA3* mRNA was decreased in adult rat testes exposed in utero to 0.1 and 10 mg/kg/day of Gen + DEHP mixtures, using microarrays, RNA-seq, and qPCR analyses. Although gene expression changes could reflect changes in the proportion of specific cell types within the tissue rather than true changes of expression in the cells, for example, a large loss of germ cells as seen in the testes of rats exposed as fetuses to Gen + DEHP with Sertoli-only phenotypes [26] could increase the proportion of Leydig cells and the representation of Leydig cell markers in the samples, this does not seem to be the case here, since FOXA3 protein was decreased, not increased, and this was observed in samples from adult rats exposed in utero to Gen + DEHP where spermatogenesis was visible. FOXA3 was originally identified in the liver as one of three members of the forkhead box A family. Forkhead box A family members are winged helix proteins that function as transcriptional regulators by binding to target sites on DNA [38]. Studies by Garon and Behr teams previously reported FOXA3 to be the only FoxA family member identified in the testes [31,32]. Here, we identified another forkhead box A member, *Foxa1*, in adult rat testes, that was downregulated (−2.33-fold change) in the RNA-seq dataset and was found to have a protein–DNA interaction with FOXA3 using a network interaction search in IPA. FOXA1 was reported to bind to the androgen receptor in the prostate. These data suggest that Foxa1 is indeed present in adult rat testes and may be altered by fetal exposure to EDCs, either directly or through interactions with Foxa3. The study identified Foxa3 to be a novel gene downregulated in adult rats by in utero exposure to environmentally relevant doses of Gen + DEHP mixtures.

Taken together, the fact that the EDC exposures took place in fetuses and that FOXA3, a gene known to be expressed in adult Leydig cells, was reduced in the testicular interstitium of adult rats exposed as fetuses to these EDCs, suggests that the developmental programming of adult type Leydig cells was disrupted in utero. Although adult Leydig cells are different from fetal Leydig cells, and differentiate from early postnatal progenitor cells that progress to immature LCs before fully differentiating to adult LCs, they have been proposed to share an undefined early fetal common precursor [39,40]. Thus, one could interpret our data to imply the disruption of a fetal precursor of adult Leydig cells jeopardizing their future differentiation in adult cell type. This is reminescent of the Developmental Origins of Health and Disease (DOHaD) theory, originally developed based on studies relating food scarcity in parents or grandparents to metabolic syndrome in children, which has been extended to many biological functions, including reproduction [11,41]. This complex phenomenon most likely involves epigenetic remodeling as well as adaptive responses of the fetus to changes in the environment which can be retained in the adult, as well as across generations [42]. However, one cannot rule out that fetal EDC exposure altered the developmental programming of other cell types in the testes, or elements of the H–P–T axis, or even other endocrine tissues interacting with testes, contributing to the effects observed in adult Leydig cells.

The search of gene targets of FOXA3 by ChIP-seq analysis identified three genes pulled down with FOXA3, *Phlpp1*, *Tmeff2,* and *Cxcl13* that have been found in testes and/or androgen-responsive tissues and may warrant further examination. PHLPP1 (PH domain and leucine-rich repeat protein phosphatase 1) has been found to be expressed in human testes, including spermatogonia [43]. TMEFF2 has also been identified in human testes, shown to prevent PDGF-AA-induced cellular proliferation, to be upregulated by androgen in some prostate cancer cells, and to have oncogenic and onco-suppressive actions depending on the tissue/cancer type context [44,45]. CXCL13 is also an androgen-responsive gene and has been shown to be involved in androgen-induced prostate cancer [46].

We also aimed to link the effect of FOXA3 downregulation in adult testes to changes in testicular function. The exact function played by FOXA3 in Leydig, Sertoli, and germ cells is not fully understood [31,32]. We used TRANSFAC, a transcription factor database, to identify FOXA3 target genes and their specific cell types. This approach pinpointed at genes associated with Leydig cells including *Hsd3b*, *Apo (A-I)*, *Pck*, *Nur77*, and *G6P.* A network interaction search on genes in our dataset related to “steroidogenesis” also found that *Tspo*, *Hsd17b1*, *Foxa1*, *Ghrh*, and a number of cytochrome P450s were downregulated. These genes are critical for the proper functioning of steroidogenesis [47,48]. A qPCR analysis further identified decreasing trends in the expression of *Cyp17*, *HSD3b*, *Cyp11a1,* and *Tspo*,which was more pronounced in adult rat testes exposed in utero to the 0.1 mg/kg/day dose. Tspo protein levels were also decreased in the interstitium, but not in seminiferous tubules, of adult rats, suggesting differential cell-specific long-term consequences of fetal EDC exposure in adult testes. TSPO is a translocator protein that plays a role in cholesterol-mediated transport from the outer to the inner mitochondrial membrane, highly expressed in and an important component of Leydig cells, but also expressed at lower levels in pachytene spermatocytes and dividing spermatogonia in adult rat testes [49,50]. These data may explain the reduction in serum testosterone levels that we previously observed in rats in utero exposed to Gen + DEHP mixtures, where the lower dose had more dramatic effects than the higher dose, further suggesting that fetal exposure to these EDC mixtures at doses lower than their individual NOAEL can affect adult steroidogenesis, a key function of the testes [26].

## 4. Materials and Methods

### 4.1. Animal Treatments and Tissue Collection

**Figure 6:**
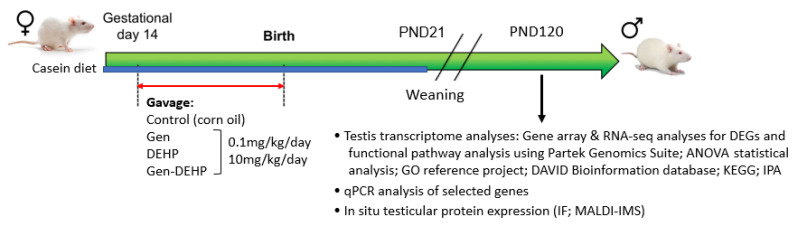
Flow chart of treatments and experiments performed.

Animal treatments and tissue collection were performed as previously described [26]. Timed pregnant Sprague-Dawley rats were purchased from Charles Rivers Laboratories (Saint-Constant, QC, Canada) and switched to a casein/cornstarch-based, phytoestrogen-free diet (casein diet) AIN-93G (Teklad diet, Envigo, Indianapolis, IN, USA) from 2 days before gavage to weaning, to avoid dietary exposure to genistein. The rats were maintained on a 12L:12D photoperiod with ad libitum access to food and water and handled according to protocols approved by the McGill University Health Centre Animal Care Committee and the Canadian Council on Animal Care. Pregnant rats were treated by gavage from gestational day 14 to parturition with either vehicle (corn oil) alone or containing GEN, DEHP, or GEN + DEHP mixtures at the doses of 0.1 and 10 mg/kg/day, encompassing exposure levels found in the general population to those measured in vegetarian/vegan women and more susceptible populations such as hospitalized neonates exposed to DEHP via medical equipment and fed soy formula (Figure 6) [16,51,52,53,54,55]. Doses were adjusted to changes in dam weights. Offspring were weighed and euthanized at PND120. The testes were collected, weighed, and either fixed in 4% paraformaldehyde or snap frozen for gene and protein expression analyses.

### 4.2. RNA Extraction and Quantitative Real-Time PCR

RNA was extracted from testes using a Nucleospin XS Kit and digested with DNase I (Takara Bio, San Jose, CA, USA). Complementary DNA was synthesized using the transcriptor synthesis kit (Roche Diagnostics, Indianapolis, IN, USA). Quantitative real-time PCR (qPCR) was performed as previously described with a LightCycler 480 using SYBR Green Supermix (BioRad, Hercules, CA, USA) and a Master Mix kit (Roche Diagnostics). Glyceraldehyde-3-phosphate dehydrogenase was used as reference to normalize gene expression. A minimum of 3–8 male offspring from different litters were assessed in triplicate. The comparative Ct method was used to calculate relative gene expression. Primers specific for the genes of interest were designed with the NCBI Primer Design Database.

### 4.3. Immunofluorescence (IF)

The IF analysis was performed as previously described [49,56]. Briefly, slides were first dewaxed and rehydrated using Citrisol and Trilogy (Cell Marque, Rocklin, CA, USA) solution. Following treatment with Dako Target Antigen Retrieval Solution (DAKO, Troy, MI, USA), the sections were incubated with PBS containing 10% BSA and 10% donkey serum for one hour to block non-specific protein interactions. The sections were then incubated with anti-forkhead box A3 (FOXA3) antibody (SAB2108468, Sigma-Aldrich, Saint Louis, MO, USA) (1:50 dilution), anti-platelet-derived growth factor receptor alpha (Pdgfrα) (sc-398206, Bioss, Woburn, MA USA) (1:50 dilution), or translocator protein (TSPO) (1:400 dilution) antibodies [57] diluted in PBS containing 10% BSA, 0.1% Triton-X, and donkey serum overnight at 4 °C. Then, the slides were incubated with a fluorescent goat anti-rabbit Alexa Fluor 488 (Thermo Fisher, Waltham, MA, USA) diluted in PBS containing 1% BSA for one hour at room temperature. Nuclear staining was performed using nuclear DAPI anti-fade and mounting medium (Vector Labs, Newark, CA, USA), coverslipped, and then imaged. Immunofluorescence signals were viewed using the appropriate filters on a Biotek Cytation 5 slide imager. The IF analysis was conducted on 2–3 independent offspring per treatment, and representative pictures are shown.

### 4.4. MALDI Imaging Mass Spectrometry

The matrix-assisted laser desorption/ionization with imaging mass spectrometry (MALDI IMS) analysis was performed by the Core Facility of the School of Pharmacy of USC, to generate “intensity maps” showing the in situ relative abundance of FOXA3 using previously described methodology [57]. Briefly, frozen PND120 adult rat testes were cryosectioned at 12 uM thickness at −21 °C and thawed on precooled ITO-coated slides. Then, sections were washed in 70% ethanol for 120 s two times, followed by washing with 100% ethanol for 120 s. The MALDI matrix consisting of sinapic acid at 10 mg/mL in 50% acetonitrile/0.1% formic acid was sprayed on sections. Matrix-coated sections were recrystallized using 50% formic acid at 80 °C for 10 min. Then, sections were imaged using a Rapiflex MALDI IMS system (Bruker, rapifleX system, Billerica, MA, USA) at 100 uM spatial resolution.

### 4.5. Microarray Analysis

The microarrays were performed as previously described [26]. Briefly, RNA was extracted with a PicoPure RNA isolation kit (Arcturus, San Diego, CA, USA) from the testes of PND120 rat offspring from three different dams per treatment. The gene array analysis was performed on Affymetrix 2.0 ST microarray chips by Genome Quebec, as previously described [56,58]. A statistical analysis was performed using the Partek Genomics Suite software, to identify differentially expressed genes (DEGs) between the Gen and DEHP treatments and control samples using ANOVA. In total, 19,786 protein-coding genes and microRNAs were analyzed. DEGs were identified using an unadjusted *p*-value of 0.05 as cutoff and applying a fold-change cutoff of at least 40% above or below the control values. The gene lists from Partek were analyzed for functional pathways and networks using the Ingenuity Pathway Analysis (IPA) software (https://www.nihlibrary.nih.gov/resources/tools/ingenuity-pathways-analysis-ipa) and the Database for Association, Visualization and Integrated Discovery (DAVID) software (https://david.ncifcrf.gov/) linked to the Kyoto Encyclopedia of Genes and Genomes (KEGG) database (https://www.genome.jp/kegg/). The 2^log2 values of the signal intensities (originally expressed as log2 values) were calculated, and genes expressed at a relative intensity below 40 (representing 33% of all genes on the arrays) in all conditions were not given priority. Gene and pathway relevance to the study were assessed via PubMed keyword searches.

### 4.6. Whole Transcriptome RNA Sequencing

Transcriptomic RNA sequencing was performed at the USC Norris Molecular Genomics Core. Total RNA were isolated using a Qiagen (Germatown, MD, USA) All prep Extraction kit following the manufacturer’s protocol (Qiagen Cat. No. 80284). Libraries were simultaneously prepared using an Illumina Truseq Stranded mRNA Library Preparation kit (Illumina Cat. No. 20020594; San Diego, CA, USA). Transcriptomic RNAseq libraries were sequenced on Illumina Nextseq500 at 25 million reads per sample at 2 × 75 read length. Data were trimmed normalized and analyzed using Partek Flow. Gene lists were created to detect differentially expressed genes between

### 4.7. ChIP-Seq Analysis

The ChIP-seq analysis was performed by Diagenode s.a. (Denville, NJ, USA) to identify potential target genes of FOXA3 in rat testes, using DNA from frozen adult control rat testes, which contains higher levels of FOXA3 protein than testes from rats exposed in utero to Gen + DEHP. Briefly, chromatin was prepared from 175 mg frozen tissue by Diagenode ChIP-seq/ChIP-qPCR Profiling service (Diagenode Cat. No. G02010000) using an iDeal ChIP-seq Kit for Transcription Factors (Diagenode Cat. No. C01010170). Chromatin shearing was performed with a Bioruptor^®^ Pico sonication device (Diagenode Cat. No. B01060001) for 4 cycles. ChIP immunoprecipitation was performed on 8 μg of chromatin using 1.5 μg and 3 μg of two anti-FOXA3 antibodies (Invitrogen PA1-813, Lot VD296799, Santa Cruz sc-74424 X, Lot A2320). Some chromatin was kept as input. ChIP efficiency was assessed by qPCR analyses, and rat primers for H3K4me3/Pol2 were used as internal ChIP controls prior to library preparation. Libraries were prepared from input and ChIP’s DNA using MicroPlex Library Preparation from Diagenode with 24 UDI for MicroPlex v3. Optimal library amplification was assessed and the libraries were purified, quantified, and fragment sizes determined. Libraries were analyzed using Illumina sequencing (NextSeq 2000 P2 200 cycles), paired-end reads, 2× 50 bp, 30 million raw reads per mark on average. Quality check, alignment to reference genome, identification of enriched regions, and annotation of ChIP-Seq peaks with genomic regions (introns, exons, promoters, 1-to-5 kb upstream-TSS, and intergenic regions) were performed.

### 4.8. Statistical Analysis

The statistical analysis was performed using one-way ANOVA with post hoc Dunnett’s test or unpaired two-tailed Student’s *t*-test for qPCR data analysis, using the statistical analysis functions in GraphPad Prism 7.04 program (GraphPad Inc. San Diego, CA, USA). Because the two EDCs used were not expected to have similar effects, an unpaired two-tailed Student’s *t*-test was used to determine the statistical significance between each control-EDC pair for qPCR analysis. The gene array analysis was performed on three independent N (one offspring/dam) per treatment condition, using the ANOVA application from the bioinformatics Partek platform. Fertility was assessed using 8 to 9 offspring from different litters per treatment condition [26]. For the qPCR analysis, the results are presented as mean ± SEM of fold changes relative to vehicle control. Experimental points were performed in triplicate for each sample, from 3 to 8 rats from different dams per treatment condition. Asterisks indicate a significant change relative to control, with *p*-values ≤0.05 considered to be statistically significant.

## 5. Conclusions

In this study, we identified functional pathways related to Leydig, Sertoli, and germ cells altered in adult rat testes following in utero exposure to mixtures of genistein and DEHP at doses encountered by humans. FOXA3, a transcription factor critical for Leydig cell function, was downregulated, as well as several genes in its interactome and steroidogenic genes. The protein expression of FOXA3 was reduced in testicular interstitium by in utero exposure to genistein + DEHP mixtures. The results of this study suggest that in utero exposure to low-dose mixtures of Gen and DEHP can disrupt the developmental program of key testicular cell types, including Leydig cells, and implies that FOXA3 is a pivotal target of the long-term adverse effects of fetal exposure to EDC mixtures on male reproduction. Our findings also suggest that FOXA3 could be used as sentinel gene to screen for potential long-term effects of fetal exposure to EDC mixtures with potential adverse male reproductive effects, in conjunction with the determination of cell-specific markers and/or morphometric analyses, to assess the possibility of effects due to changes in cellularity rather than cell-specific gene/protein alterations.

## Figures and Tables

**Table 1:** In utero exposure to a 10 mg/kg/day Gen + DEHP mixture affects Sertoli and germ cell pathways in adult offspring. KEGG analysis of differentially expressed genes (DEGs) by microarray analysis reveals canonical pathways related to Sertoli and germ cells in rat testes exposed in utero to 10 mg/kg/day of Gen + DEHP. Statistical cut-offs of 40% fold change with an unadjusted *p*-value of 0.05 were used to obtain gene lists. Three to four pups were used per treatment.

Term	Gene Count	Percentage	*p*-Value
Hippo signaling pathway	16	1.6	0.0041
Retinol metabolism	11	1.1	0.0042
Wnt signaling pathway	14	1.4	0.0120
cGMP-PKG signaling pathway	15	1.5	0.0160
Adherens junction	9	0.9	0.0170
Galactose metabolism	5	0.5	0.0560
Metabolic pathways	70	6.9	0.0740
MAPK signaling pathway	18	1.8	0.0770
Oxytocin signaling pathway	22	1.2	0.0860

**Table 2:** FOXA3 is the most downregulated DEG in adult rat testes exposed in utero to a 10 mg/kg/day Gen + DEHP mixture. The KEGG analysis of microarrays shows the 10 most downregulated (green) and 10 most upregulated (red) genes.

Symbol	Entrez Gene Name	Fold Change	*p*-Value
*Foxa3*	forkhead box A3	−2.609	0.0001
*Tmem249*	transmembrane protein 249	−2.439	0.0412
*Krt86*	keratin 86	−2.397	0.0381
*Tmem210*	transmembrane protein 210	−2.378	0.0420
*Ldoc1*	LDOC1 regulator of NFKB signaling	−2.279	0.0109
*Pkmyt1*	protein kinase, membrane associated tyrosine/threonine 1	−2.214	0.0053
*Olr1749*	olfactory receptor 1749	−2.201	0.0054
*Cyp2g1*	cytochrome P450, family 2, subfamily g, polypeptide 1	−2.183	0.0332
*Krtap10-7*	keratin associated protein 10-7	−2.149	0.0060
*Or1f1*	olfactory receptor family 1 subfamily F member 1	−2.124	0.0010
*Gpr34*	G protein-coupled receptor 34	2.497	0.0192
*Sat1*	spermidine/spermine N1-acetyltransferase 1	2.549	0.0319
*Scarb2*	scavenger receptor class B member 2	2.551	0.0362
*St3gal4*	ST3 beta-galactoside alpha-2,3-sialyltransferase 4	2.611	0.0393
*Xiap*	X-linked inhibitor of apoptosis	2.64	0.0357
*Abca6*	ATP binding cassette subfamily A member 6	2.719	0.0496
*Clic2*	chloride intracellular channel 2	2.728	0.0448
*Cyp11a1*	cytochrome P450 family 11 subfamily A member 1	2.865	0.0419
*Cyp2a6*	cytochrome P450 family 2 subfamily A member 6	3.128	0.0235
*Cyp2a1*	cytochrome P450, family 2, subfamily a, polypeptide 1	4.044	0.0410

**Table 3:** In silico search for FOXA3-interacting genes in testes. The IPA database was searched for genes with a relationship to ”FOXA3” in adult rat testes exposed in utero to (**A**) 0.1 and (**B**) 10 mg/kg/day Gen + DEHP mixtures using the IPA of RNA-seq data. Data are shown in fold change of control samples. *N* = 4 per treatment, *p* ≤ 0.05, FC = 2; (**C**) TRANSFAC-generated list of FOXA3 target genes in testes.

**A**			
**Symbol**	**Gene Name**	**Fold Change**	***p*-Value**
*Cox4I2*	cytochrome c oxidase subunit 4I2	−4.026	0.000
*Zbtb33*	zinc finger and BTB domain containing 33	−3.544	0.008
*Foxa1*	forkhead box A1	−2.333	0.007
*Nrob2*	nuclear receptor subfamily 0 group B member 2	−2.076	0.004
*Khdrbs2*	KH RNA binding domain containing, signal transduction associated 2	−2.021	0.028
*Lmnb2*	lamin B2	2.164	0.001
**B**			
**Symbol**	**Gene Name**	**Fold Change**	***p*-Value**
*Foxa3*	forkhead box A3	−2.609	0.000
*Klhl13*	kelch like family member 13	1.408	0.036
*P4ha1*	prolyl 4-hydroxylase subunit alpha 1	1.492	0.044
*Nsf*	N-ethylmaleimide sensitive factor, vesicle fusing ATPase	1.705	0.050
*Cycs*	cytochrome c, somatic	1.971	0.026
**C**			
**Symbol**	**Gene Name**	**Testis Cell Type(s)**	**Relation to Foxa3**
*Hoxa10*	Homeobox protein A10	Leydig	Target gene
*Hsd3β*	Hydroxysteroid dehydrogenase 3 β	Leydig	Target gene
*Apo(a-I)*	Apolipoprotein (A-I)	Leydig	Target genes *(ApoE)* In Foxa2/Foxa3 TF network
*Pck-1*	Phosphoenolpyruvate carboxy kinase	Leydig (Steroidogenesis)	Target gene In Foxa2/Foxa3 TF network
*Hmgb1*	High mobility group box 1	Sertoli, germ	Protein-protein interaction
*Pparα*	Peroxisome proliferator receptor alpha	Sertoli, germ, Leydig	Protein-protein interaction
*G6p*	Glucose-6-phosphatase	Leydig (steroidogenesis)	In Foxa2/Foxa3 TF network
*Nur77*	Nuclear receptor subfamily 4 group A member 1	Leydig (Steroidogenesis)	Target gene
*Tf*	Transferrin	Sertoli	Target gene
*Tle3*	Transducin like enhancer of split 3	Sertoli	Target gene

**Table 4:** Fetal exposure to Gen + DEHP mixtures affects steroidogenesis-related genes. Differentially expressed genes related to “steroidogenesis” in adult rat testes exposed to 0.1 mg/kg/day Gen + DEHP mixture generated from ingenuity pathway analysis of RNA-seq data. Data are shown in fold change of control samples. *N* = 4 per treatment, *p* ≤ 0.05, FC = 2.

Symbol	Steroidogenesis-Related Gene Name	Fold Change	*p*-Value
*Ghrh*	growth hormone releasing hormone	−2.690	0.008
*Foxa1*	forkhead box A1	−2.333	0.007
*Hsd17b1*	hydroxysteroid 17-beta dehydrogenase 1	−2.272	0.032
*Cypb1*	cytochrome P450 family 7 subfamily B member 1	−2.260	0.020
*Cyp7a1*	cytochrome P450 family 7 subfamily A member 1	−2.164	0.018
*Cyp2r1*	cytochrome P450 family 2 subfamily R member 1	−2.066	0.005
*Tspo*	translocator protein	−2.047	0.012
*Apoa1*	apolipoprotein A1	2.080	0.002

**Table 5:** Annotation table of genes identified by ChIP-seq as binding to FOXA3. In total, 18 protein coding genes and one ribosomal RNA were identified after sequencing the libraries of DNA fragments pulled down using 1.5 or 3 µg of two anti-FOXA3 antibodies. A ChIP-seq analysis was performed on DNA from frozen adult control rat testes. Distance to TSS, nearest promoter ID, and gene name are indicated. Several non-coding sequences were removed from the table.

Antibody	Peak ID	Chromosome	Start	End	Strand	Annotation	Detailed Annotation	Distance to TSS	Nearest Promoter ID	Gene Name	Gene Description	Gene Type
Invitrogen 1.5 ug	001_0788_0 01_peak_1	chrY	1841330	1841557	+	Intergenic	SATI_RN|Satellite|Satellite	−438021	NM_001329902	Usp9y	ubiquitin specific peptidase 9, Y-linked	protein-coding
Invitrogen 3 ug	001_0788_0 02_peak_7	chr20	27473893	27474238	+	Intergenic	Intergenic	78160	NM_147146	Rwdd1	RWD domain containing 1	protein-coding
	001_0788_0 02_peak_5	chr14	15463091	15463463	+	Intergenic	Lx2B|LINE|L1	−205092	NM_001017496	Cxcl13	C-X-C motif chemokine ligand 13	protein-coding
	001_0788_0 02_peak_2	chr1	147551697	147552007	+	intron (intron 9)	RNHAL1|LINE|L1	−162049	NM_001013904	Cyp2c6v1	cytochrome P450, CYP2C6, variant 1	protein-coding
	001_0788_0 02_peak_8	chr5	91563314	91563577	+	Intergenic	Lx2B|LINE|L1	428461	NR_046238	Rn5-8s	5.8S ribosomal RNA	rRNA
	001_0788_0 02_peak_9	chr7	48411144	48411477	+	Intergenic	Intergenic	−138146	NM_001108745	Ppfia2	PTPRF interacting protein alpha 2	protein-coding
	001_0788_0 02_peak_3	chr1	233681071	233681328	+	Intergenic	Lx2A|LINE|L1	298421	NM_031036	Gnaq	G protein subunit alpha q	protein-coding
	001_0788_0 02_peak_4	chr10	74769530	74769818	+	Intergenic	Intergenic	−45727	NM_001129777	Rad51c	RAD51 paralog C	protein-coding
SantaCruz 1.5 ug	001_0788_0 03_peak_9	chr20	27473924	27474228	+	Intergenic	Intergenic	78149	NM_147146	Rwdd1	RWD domain containing 1	protein-coding
	001_0788_0 03_peak_3	chr15	92078631	92078967	+	Intergenic	ERVB2_2-I_RN|LTR|ERVK	1228621	NM_001107283	Slitrk1	SLIT and NTRK-like family, member 1	protein-coding
	001_0788_0 03_peak_6	chr2	132257139	132257414	+	Intergenic	Intergenic	−1599074	NM_001106429	Pabpc4l	poly(A) binding protein, cytoplasmic 4-like	protein-coding
	001_0788_0 03_peak_13	chr9	114715355	114715651	+	Intergenic	Intergenic	−5890	NM_013017	Rab12	RAB12, member RAS oncogene family	protein-coding
	001_0788_0 03_peak_10	chr6	89503088	89503335	+	Intergenic	Intergenic	−211184	NM_199269	Mdga2	MAM domain containing glycosylphosphatidylinositol anchor 2	protein-coding
	001_0788_0 03_peak_12	chr9	56664665	56664957	+	Intergenic	Lx2|LINE|L1	−991107	NM_001108795	Tmeff2	transmembrane protein with EGF-like and two follistatin-like domains 2	protein-coding
	001_0788_0 03_peak_2	chr13	109148367	109148629	+	Intergenic	Intergenic	−307016	NM_001107200	Ptpn14	protein tyrosine phosphatase, non-receptor type 14	protein-coding
	001_0788_0 03_peak_4	chr15	103206833	103207086	+	Intergenic	Intergenic	133280	NM_001107286	Tgds	TDP-glucose 4,6-dehydratase	protein-coding
	001_0788_0 03_peak_7	chr2	173864996	173865243	+	Intergenic	Lx6|LINE|L1	147557	NM_001009542	Pdcd10	programmed cell death 10	protein-coding
	001_0788_0 03_peak_11	chr8	125968054	125968301	+	Intergenic	RNLTR8C2|LTR|ERVK	−443652	NM_001025705	Azi2	5-azacytidine induced 2	protein-coding
SantaCruz 3 ug	001_0788_0 04_peak_5	chr20	27473930	27474176	+	Intergenic	MamRTE1|LINE|RTE-BovB	78172	NM_147146	Rwdd1	RWD domain containing 1	protein-coding
	001_0788_0 04_peak_3	chr19	58032198	58032458	+	Intron (intron 10)	B4A|SINE|B4	212068	NM_175596	Disc1	DISC1 scaffold protein	protein-coding
	001_0788_0 04_peak_2	chr13	26223458	26223722	+	intron (intron 4)	intron (intron 4)	51375	NM_021657	Phlpp1	PH domain and leucine rich repeat protein phosphatase 1	protein-coding
	001_0788_0 04_peak_7	chr8	53458384	53458659	+	Intergenic	Intergenic	47205	NM_153311	Tmprss5	transmembrane serine protease 5	protein-coding
	001_0788_0 04_peak_6	chr20	39622048	39622391	+	Intergenic	RNLTR3d|LTR|ERVK	−614218	NM_001106392	Hs3st5	heparan sulfate-glucosamine 3-sulfotransferase 5	protein-coding

**Table 6:** mRNA expression changes in genes identified by ChIP-seq as binding to FOXA3. The 18 genes found to bind FOXA3 by ChIP-seq analysis of an adult control testis sample were examined in the RNA-seq data of control rats vs. in utero Gen + DEHP-exposed rat testes.

	GD 0.1 vs. Ctrl		GD 10 vs. Ctrl	
Gene	Fold Change	*p* Value	Fold Change	*p* Value
*Cxcl13*	−1.36	0.605	−1.04	0.209
*Mdga2*	−1.14	0.916	1.05	0.277
*Gnaq*	−1.13	0.178	−1.12	0.951
*Pabpc4*	−1.08	0.384	1.10	0.106
*Rn5-8s*	−1.06	0.845	−1.02	0.745
*Ppfia2*	−1.06	0.435	−1.07	0.920
*Rab12*	−1.05	0.635	1.04	0.280
*Tgds*	−1.02	0.473	1.11	0.338
*Usp9y*	1.02	0.276	1.17	0.234
*Ptpn14*	1.04	0.422	1.05	0.859
*Tmprss5*	1.04	0.952	−1.49	0.891
*Rad51c*	1.05	0.318	−1.08	0.116
*Pdcd10*	1.07	0.728	−1.02	0.271
*Rwdd1*	1.08	0.904	1.05	0.720
*Azi2*	1.15	0.493	−1.04	0.00757
*Cyp2c6v1*	1.16	0.221	1.11	0.585
*Phlpp1*	1.20	0.0244	1.68	0.110
*Tmeff2*	1.50	0.347	1.55	0.249

## Data Availability

The data supporting reported results can be found in the manuscript.

## Supplementary Materials

The following supporting information can be downloaded at: https://www.mdpi.com/article/10.3390/ijms24021211/s1.
