# Supplementary material for: Impact of Fetal Exposure to Endocrine Disrupting Chemical Mixtures on FOXA3 Gene and Protein Expression in Adult Rat Testes
Source: Int J Mol Sci. 2023 Jan 7;24(2):1211. doi: 10.3390/ijms24021211 (PMC9863867; doi:10.3390/ijms24021211)
Supplement: Supplementary file 1 [file ijms-24-01211-s001.zip › ijms-2039238-supplementary.pdf]

Supplemental Tables (**ijms-2039238)**

Supplemental Table S1. Fetal exposure to Gen or DEHP alone does not alter Foxa3-related genes that were affected by exposure to their mixtures in adult testes. *In Silico* IPA search of Foxa3 interacting-genes in testes of adult rats exposed in utero to vehicle, or 10 mg/kg/day of Gen or DEHP alone, was performed as explained in Table 3, which examined differentially expressed genes in response to the mixtures, except that only the 10 mg/kg/day dose was analyzed for individual EDCs by RNA-seq. Data are shown in fold change of control samples. N=4 per treatment. p≤0.05, FC=2.

Foxa3-interacting genes				
	Gen vs. Control		DEHP vs. Control	
Gene symbol	P-value	Fold change	P-value	Fold change
Cox4i2	2.26E-01	-1.75	8.30E-02	-2.78
Zbtb33	6.02E-01	-1.34	4.45E-01	-1.43
Foxa1	7.31E-01	-1.23	3.03E-01	-1.77
Khdrbs2	7.05E-01	-1.27	3.12E-01	-2.07
Lmnb2	7.56E-02	1.49	3.65E-01	1.26
Klhl13	6.30E-01	-1.05	6.48E-01	-1.08
P4ha1	2.42E-01	-1.17	7.25E-01	1.05
Nsf	9.23E-01	-1.01	8.42E-01	1.02
Cycs	7.05E-01	-1.03	2.34E-01	1.13
Hmgb1	9.93E-01	1.00	4.61E-02	1.22
Ppara	4.37E-01	1.19	9.11E-01	1.01
Tle3	8.11E-01	1.03	9.45E-01	-1.01

Supplemental Table S2. Fetal exposure to Gen or DEHP alone does not alter genes that were affected by exposure to their mixtures. *In Silico IPA* search for genes related to steroidogenesis in testes of adult rats exposed in utero to 10 mg/kg/day of Gen or DEHP individually, was performed as described in Table 4, that reported differentially expressed genes related to 'steroidogenesis' in rats exposed to Gen or DEHP mixtures, by RNA-seq analysis. Data are shown in fold change of control samples. N=4 per treatment. p≤0.05, FC=2.

Steroidogenesis-related genes				
	Gen vs. Control		DEHP vs. Control	
Gene symbol	P-value	Fold change	P-value	Fold change
Ghrh	7.70E-01	1.01	8.25E-01	-1.09
Foxa1	7.31E-01	-1.23	3.03E-01	-1.77
Hsd17b1	7.20E-01	-1.16	3.92E-01	-1.55
Cyp7b1	3.00E-01	-1.53	5.90E-01	-1.28
Cyp2r1	6.24E-01	-1.15	1.98E-01	-1.62
Cyp7a1	7.45E-01	-1.22	4.71E-01	-1.25
Tspo	9.05E-01	1.06	1.86E-01	-1.67
Apoa1	1.98E-01	1.37	2.24E-01	1.38

Supplemental Table S3. Fetal exposure to Gen or DEHP alone does not alter the expression of genes found to co-precipitate with Foxa3 protein by Chip-seq analysis. The 18 genes found to bind Foxa3 by ChIP-seq analysis of an adult control testis sample (Table 6) were examined in the RNA-seq data of adult rats exposed in utero to vehicle or 10mg/kg/day of either Gen or DEHP alone.

Genes identified by Foxa3 Chip-seq analysis				
10 mg/kg/day	**Gen vs. Control**		**DEHP vs. Control**	
**Gene symbol**	**P-value**	**Fold change**	**P-value**	**Fold change**
Cxcl13	6.93E-01	-1.15	9.67E-01	-1.03
Mdga2	6.98E-01	1.10	2.61E-01	-1.26
Gnaq	1.15E-01	1.34	3.73E-01	1.21
Pabpc4	6.22E-01	1.04	5.92E-01	1.05
Rn5-8s	6.29E-01	1.16	9.33E-01	-1.13
Ppfia2	4.20E-01	-1.12	2.59E-01	1.21
Rab12	2.17E-01	-1.12	9.43E-01	1.00
Tgds	2.84E-01	-1.17	8.62E-01	1.02
Usp9y	3.37E-01	1.09	4.30E-01	1.09
Ptpn14	1.38E-01	1.31	9.58E-02	1.43
Tmprss5	3.98E-01	1.28	6.51E-01	-1.49
Rad51c	2.53E-01	-1.14	5.20E-01	-1.10
Pdcd10	7.56E-01	-1.02	3.89E-01	-1.19
Rwdd1	3.35E-01	-1.11	1.57E-01	-1.21
Azi2	2.54E-01	-1.09	2.83E-01	-1.11
Cyp2c6v1	3.19E-01	-1.41	5.24E-02	-2.47
Phlpp1	3.42E-02	1.22	4.49E-01	1.08
Tmeff2	2.71E-01	-1.15	3.01E-01	1.16
